# Type A Thoracic Aortic Dissection Following Endovascular Repair of a Common Iliac Artery Aneurysm

**DOI:** 10.7759/cureus.13971

**Published:** 2021-03-18

**Authors:** Matthew P Wolfers, Qian Yu, Lisandro Montorfano, Stephen J Bordes, Terry King

**Affiliations:** 1 General Surgery, Cleveland Clinic Florida, Weston, USA; 2 Surgical Anatomy, Tulane University School of Medicine, New Orleans, USA; 3 Vascular Surgery, Cleveland Clinic Florida, Weston, USA

**Keywords:** aortic dissection, evar, endovascular aneurysm repair, aortic valve replacement, type a acute aortic dissection

## Abstract

We discuss a rare case of acute Type A thoracic aortic dissection (TAAD) following endovascular aneurysm repair (EVAR) of a common iliac artery aneurysm, which likely resulted from complications due to aberrant anatomy. Valve replacement, ascending aortic arch graft, and entry tear suture repair were necessary to contain the TAAD. Postoperative computed tomography with angiography (CTA) demonstrated stable disease, and the patient remained asymptomatic. Open and endovascular repair of the descending abdominal aorta was avoided. Few cases in the literature report TAAD following EVAR. Detection and repair of the entry site was crucial for containing the TAAD.

## Introduction

Isolated iliac artery aneurysms (IAA) are less prevalent than abdominal aortic aneurysms (AAA), though these two etiologies occasionally co-exist [1]. According to the European Society for Vascular Surgery, most IAA can be managed conservatively unless the patient is symptomatic or if the diameter of the aneurysm is greater than 3.5 cm [2]. Endovascular aneurysm repair (EVAR) has been proven to be an effective and less invasive treatment compared to open surgery for both AAA and IAA [3]. Nevertheless, EVAR is associated with complications such as endoleak, graft migration, infection, ischemia due to vessel occlusion, and access site hematoma [4]. Rarely, aortic dissections have been documented following EVAR, leading to graft collapse, impending rupture of an AAA, and distal malperfusion. The majority of these cases were Type B aortic dissections (TBAD) [5]. The present case report describes a Type A aortic dissection (TAAD) following endovascular repair of an isolated external IAA, which required open sternotomy with aortic valve replacement (AVR) and aortic graft to seal the entry tears.

## Case presentation

**Figure 1:**
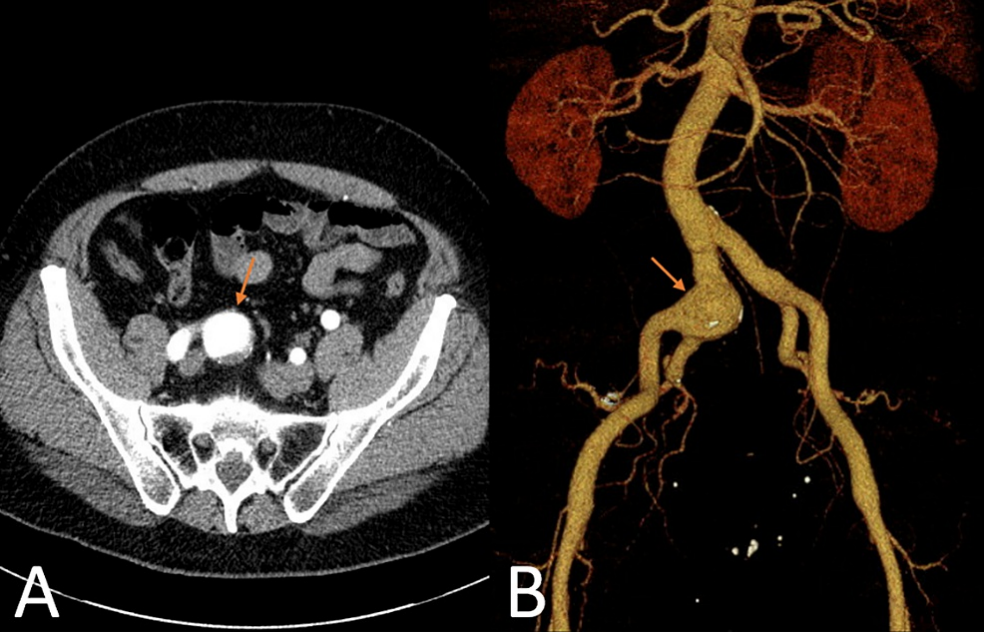
Preoperative Computed Tomography (CT) Imaging 3.7 cm fusiform aneurysm of the right common iliac artery bifurcation with slight peripheral thrombosis. The aneurysm propagates into the proximal aspect of the right internal iliac artery. (A) axial abdominal CT image. (B) Coronal abdominal CT image with three-dimensional (3D) reconstruction. Orange arrow: common iliac artery aneurysm.

A 63-year-old man with a history of atrial fibrillation, stage III chronic kidney disease, osteoarthritis, and hyperlipidemia was referred to vascular surgery for right groin pain. Computed tomography with angiography (CTA) revealed a fusiform aneurysm with a diameter of 3.7 cm in the right common iliac artery bifurcation (Figures 1A, 1B). The decision was made to proceed with endovascular treatment by deploying an iliac branch endoprosthesis. Given the proximity of the aneurysm to the aortic bifurcation, an additional aorto-iliac main body endograft was required to secure the iliac endograft and provide an adequate seal to the aneurysmal sac.

The procedure was performed using the standard bilateral femoral artery cutdown approach. With the help of a Lunderquist wire (Cook Medical, Bloomington, IN, USA) and 16 French (Fr) Gore-Tex sheath (WL Gore & Associates, Flagstaff, AZ, USA), a 23x14x10 mm Gore right iliac bell-bottom device (WL Gore & Associates, Flagstaff, AZ, USA) was deployed in the right common iliac artery, whereas the main aorto-iliac body (Gore Excluder aortoiliac prosthesis, 23x12x10 mm, WL Gore & Associates, Flagstaff, AZ, USA) was delivered on the left side, below the renal artery origins. Accordingly, the iliac branch endograft was deployed in the right iliac artery. Because the hypogastric artery was stenosed, balloon angioplasty was performed with a 6-mm balloon to provide access. A Gore Viabahn VBX stent graft (WL Gore & Associates, Flagstaff, AZ, USA) was delivered with an Amplatz wire (BostonScientific, MA, USA) into the hypogastric artery with a landing zone in the iliac branch endograft. The right graft limb of the iliac branch graft was deployed in the external iliac artery. Patency was verified throughout the graft system without evidence of an endoleak on completion angiogram.

**Figure 2:**
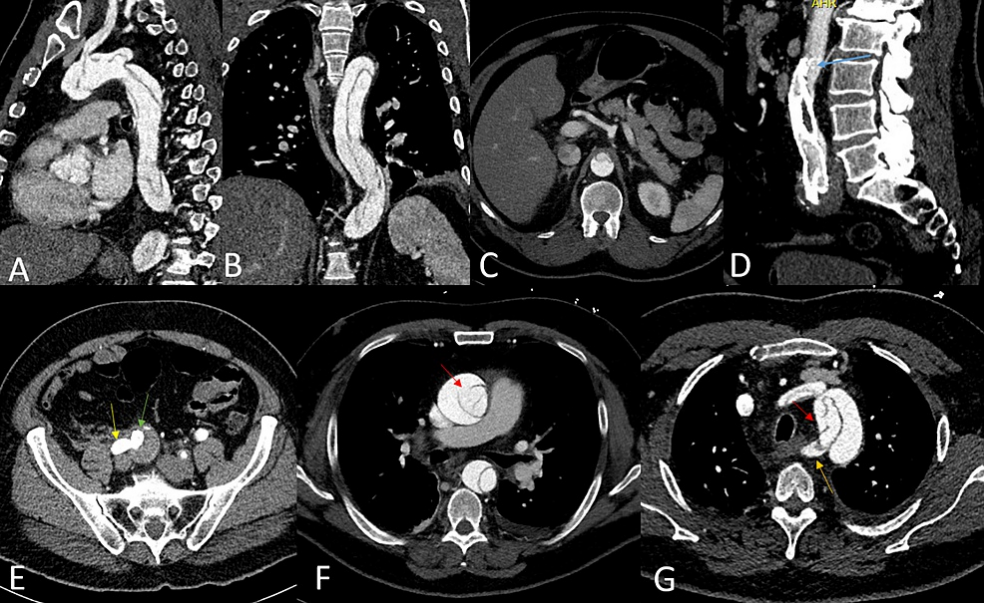
Postoperative Computed Tomography (CT) Imaging An acute aortic dissection extends from the proximal thoracic aorta (A) to the abdominal aorta (B), just below the level of the superior mesenteric artery (C). Dissection flap extends to the bilateral common carotid and subclavian arteries. The proximal end of the endograft was partially collapsed due to the dissection (D). No endoleak or dissection was noted in the right iliac artery aneurysm (E). Proximal ascending aorta (F) and right subclavian origin (G) entry tears seemed to correspond to intraoperative findings. Red arrows: entry tears. Yellow arrow: external iliac artery. Green arrow: internal iliac artery. Blue arrow: collapsed proximal endograft. Orange arrow: aberrant right subclavian artery. (A) Sagittal thoracic CT image. (B) Coronal thoracic and abdominal CT image. (C) Axial abdominal CT image. (D) Sagittal lumbar CT image. (E) Axial abdominal CT image. (F,G) Axial thoracic CT image.

**Figure 3:**
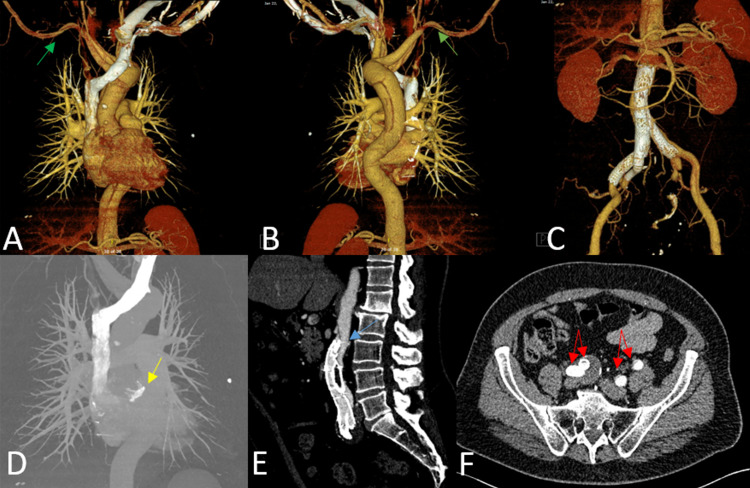
Six-Month Follow-Up Computed Tomography (CT) Imaging CT imaging of the thorax showing aberrant right subclavian artery (green arrow) branching off from the distal aortic arch with a retroesophageal course. (A) Anterior thoracic CT image with three-dimensional (3D) reconstruction. (B) Posterior thoracic CT image with 3D reconstruction. (C) 3D anterior abdominal CT imaging showing a bifurcated stent graft of the right common iliac artery excluding the right common iliac artery aneurysm. (D) Coronal thoracic CT image showing the aortic valve in place (yellow arrow). (E) Sagittal lumbar CT image showing that the proximal end of the endograft remained partially collapsed (blue arrow). (F) Axial abdominal CT showing no evidence of endoleak in the right iliac artery. Red arrows: bilateral internal and external iliac arteries opacified by contrast.

Two days later, the patient complained of right thigh numbness and right flank discomfort which was accompanied by decreased pedal pulses. CTA showed TAAD dissection from the ascending aorta into the proximal abdominal aorta below the level of the superior mesentery artery origin, compressing the proximal aspect of the stent (Figures 2A-2C). The right limb of the endograft was patent, though collapse was observed on the left (Figures 2D, 2E). The dissection flap extended to the bilateral common carotid and subclavian arteries. Vitals were within normal limits, but echocardiogram showed aortic valve regurgitation. Cardiothoracic surgery was consulted for TAAD open surgical repair and AVR with hypothermic circulatory arrest. In the operating room, severe aortic insufficiency was noted, and the aortic valve was replaced with a 23-mm Trifecta valve. There was a tear in the ascending aorta above the level of the sinutubular junction (Figure 2F). The ascending aortic arch was resected, and the residual dissection was repaired using interrupted Prolene sutures reinforced with felt strips. A 28-mm Hemashield platinum graft (Boston Scientific, Natick, MA, USA) was used to reconstruct the aorta. Prior to the EVAR, the patient had a known vascular ring with anomalous origin of the right subclavian artery from the descending aorta with a retroesophageal course. Another intimal tear was noted in the distal arch between the origins of the left carotid artery and the aberrant right subclavian artery, which was sutured accordingly (Figure 2G). Surgery was uncomplicated, and the patient had an uneventful postoperative recovery. He regained distal pedal pulses and was able to ambulate. At his six-month follow-up, the dissection remained stable (Figures 3A-3F). No evidence of an endoleak or stent-graft migration was observed. Perfusion to distal extremities was adequate despite the partially collapsed endograft. The patient’s groin pain resolved, and his recovery remained uncomplicated 18 months later.

## Discussion

We discuss a case of acute TAAD following abdominal EVAR of a common IAA. Due to the patient’s aortic insufficiency, proximal entry site, and aberrant anatomy, the TAAD was successfully treated with valve replacement, ascending aortic arch graft, and entry tear suture repair.

Based on the German Registry for Acute Aortic Dissection Type A (GERAADA), iatrogenic TAAD most commonly resulted from cardiac surgery (43.0%) and endovascular cardiac interventions (40.0%), while aortic endovascular interventions only accounted for 7% of all reported cases [6]. In a meta-analysis of iatrogenic TAAD, the percentage of patients who had cardiac surgery, cardiac catheterization, and thoracic endovascular aortic repair (TEVAR) were 89%, 7%, and 4%, respectively [7]. Although no intrinsic patient characteristics were determined to be significant predictors of mortality, individuals who underwent surgical repair and individuals who were diagnosed intraoperatively were associated with higher survival [7]. Most data on EVAR-related TAAD derived from TEVAR, which was found to have a postoperative TAAD incidence as high as 6.8%. Technical factors of such complication include intimal tears from wire manipulation, endografts rubbing the aortic wall, repeated balloon remodeling, and oversized endografts [8]. In the present case, the most likely etiology was intimal wall trauma from the wire. The distal tear between the ectopic right and left subclavian arteries was likely caused by the distal Lunderquist wire passed through the femoral access site. Meanwhile, the proximal tear in the ascending aorta may have been caused during attempted cannulation of the descending aorta from the brachial artery access site. The patient’s aberrant anatomy may have resulted in passage of the wire toward the ascending aorta. However, ballooning of the proximal fixation site may have also caused a retrograde dissection.

The majority of reported aortic dissections following EVAR are Type B [9-17]. TEVAR was commonly implemented to enclose the dissection entry point, achieving promising clinical efficacy. Similar to the present case, the distal site of the left subclavian origin along the aorta is a common entry point for dissection, which could be related to wire manipulation from femoral and brachial artery access sites [9-17]. In the present case, the patient’s immediate postoperative TAAD was symptomatic, requiring immediate open surgery through sternotomy to seal the entry tear.

To our knowledge, only one TAAD after EVAR has been previously described in the literature. In that case, TAAD occurred eight years after an EVAR for an AAA and recent endovascular Type IB endoleak repair through brachial artery access [5]. CTA showed iatrogenic TAAD extending from the ascending aorta into the AAA, which continued to grow despite emergent hemiarch replacement and TEVAR. As such, an open surgical repair with a Dacron graft sewn to the prior EVAR was performed and successfully treated this complication. In the present case, the TAAD was successfully managed with hemiarch and AVR only. The patient had no AAA and the dissection remained stable on follow-up CTA, requiring no further endovascular or surgical treatment. Open abdominal surgery was avoided as the entry point was obliterated by the initial open repair of the entry site.

## Conclusions

Acute TAAD is a rare, but major complication following EVAR of isolated IAA. Physicians should be cognizant of such complication and aim to repair the entry tear to prevent further dissection. Additional clinical follow-up is necessary to provide recommendations regarding a partially compressed endograft.
